# Sclerosing Intracancellous Osteoid Osteoma Presenting as a Painless Swelling of the Middle Phalanx in the Toe of a 15-Year-Old Footballer

**DOI:** 10.7759/cureus.82466

**Published:** 2025-04-17

**Authors:** Nikolaos Laliotis, Panagiotis Konstantinidis, Chrysanthos Chrysanthou, Anestis Moumtzouoglou, Lamprini Giannakopoulou, Katerina Zarampouka

**Affiliations:** 1 Orthopedics, Interbalkan Medical Center, Thessaloniki, GRC; 2 Orthopedics and Traumatology, Interbalkan Medical Center, Thessaloniki, GRC; 3 Anatomy and Surgical Anatomy, Aristotle University of Thessaloniki, Thessaloniki, GRC; 4 Radiology, Interbalkan Medical Center, Thessaloniki, GRC; 5 Pathology, Istodierevnitiki SA, Thessaloniki, GRC

**Keywords:** bening bone tumor, bening tumor, foot tumors, intracancellous, osteiod osteoma, osteoid tumor, painless toe swelling, toe

## Abstract

Osteoid osteoma (OO) most commonly affects the cortices of long bones and rarely involves the toes, where it typically presents with swelling and pain. Radiological evaluation can often pose a diagnostic challenge. We report the case of a boy with a painless swelling of the middle phalanx of the third toe. Radiographic assessment using plain X-rays, MRI, and CT revealed a centrally located sclerotic lesion with a barely visible nidus. A bone scan demonstrated increased uptake at the lesion site. Surgical intervention was performed to remove the sclerotic lesion, followed by reconstruction of the phalanx using an autologous bone graft. Histopathological analysis confirmed the diagnosis of OO. Although OO of the toes is uncommon, it may present as painless swelling. A centrally located sclerotic lesion in the phalanx warrants thorough investigation and surgical management with pathological confirmation to ensure accurate diagnosis.

## Introduction

Osteoid osteoma (OO) is the most common benign lesion affecting long bones, typically occurring in individuals during the second and third decades of life. These lesions are usually found in the cortical regions of long bones; however, rare cases involving intramedullary locations in the bones of the hand and foot have been reported. Involvement of the bones of the foot has been documented, with phalangeal lesions accounting for less than 2% of all OO cases [1-3]. OOs located in the toe phalanges are extremely rare, with only a few cases described in the literature [4-6].

Pain is the hallmark symptom of OO, initially intermittent and progressively worsening, often intensifying at night. The pain typically responds well to anti-inflammatory medications, which act on the elevated prostaglandin levels associated with the lesion. Toe swelling is another commonly reported clinical sign in OO cases involving the toes [2-5].

We report the case of a 15-year-old adolescent who presented with swelling of the middle toe on his left foot. An active football trainee, he initially attributed the unusual toe shape to a possible sports-related injury. Plain X-rays revealed a sclerotic bony island in the medullary region of the middle phalanx of the third toe, surrounded by a radiolucent rim. Further diagnostic evaluation - including MRI, CT scan, and bone scintigraphy - suggested an OO; however, differential diagnoses such as infection or intramedullary calcification remained considerations due to the sclerosing nature of the lesion.

Although radiofrequency (RF) ablation is our preferred treatment modality for OO, we opted for excisional biopsy in this case to establish a definitive diagnosis. The defect was reconstructed using an autologous bone graft. Histopathological examination confirmed the diagnosis of OO. The patient made a full recovery.

## Case presentation

A 15-year-old adolescent boy, actively participating in football, noticed swelling in the middle toe of his left foot over the past three months. However, he could not recall any specific injury to the toe and suspected it might be due to an unnoticed injury. He experienced discomfort while kicking the ball and reported mild pain during prolonged walking over the last 10 days, but he did not experience any night pain. He appeared to be in good health, with no swelling in any other part of his body.

**Figure 1:**
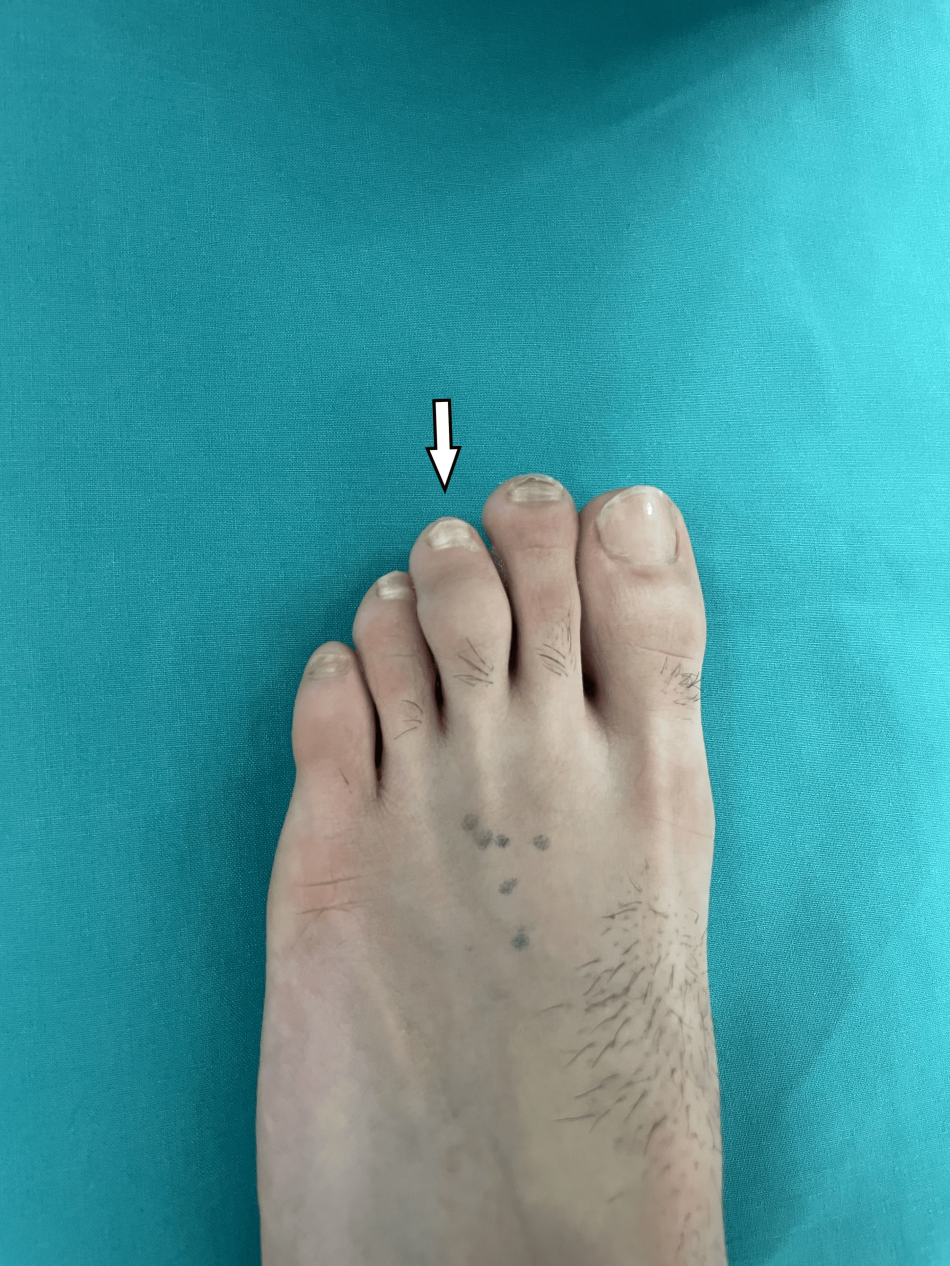
Swelling of the middle phalanx of the third toe, with normal skin coloration

He was initially referred to his local hospital, where an X-ray was performed, and then he attended our pediatric orthopedic department. On examination, the toe appeared swollen, with normal skin color and temperature. There was minimal limitation of movement in the interphalangeal joints, likely due to the swelling (Figure 1).

**Figure 2:**
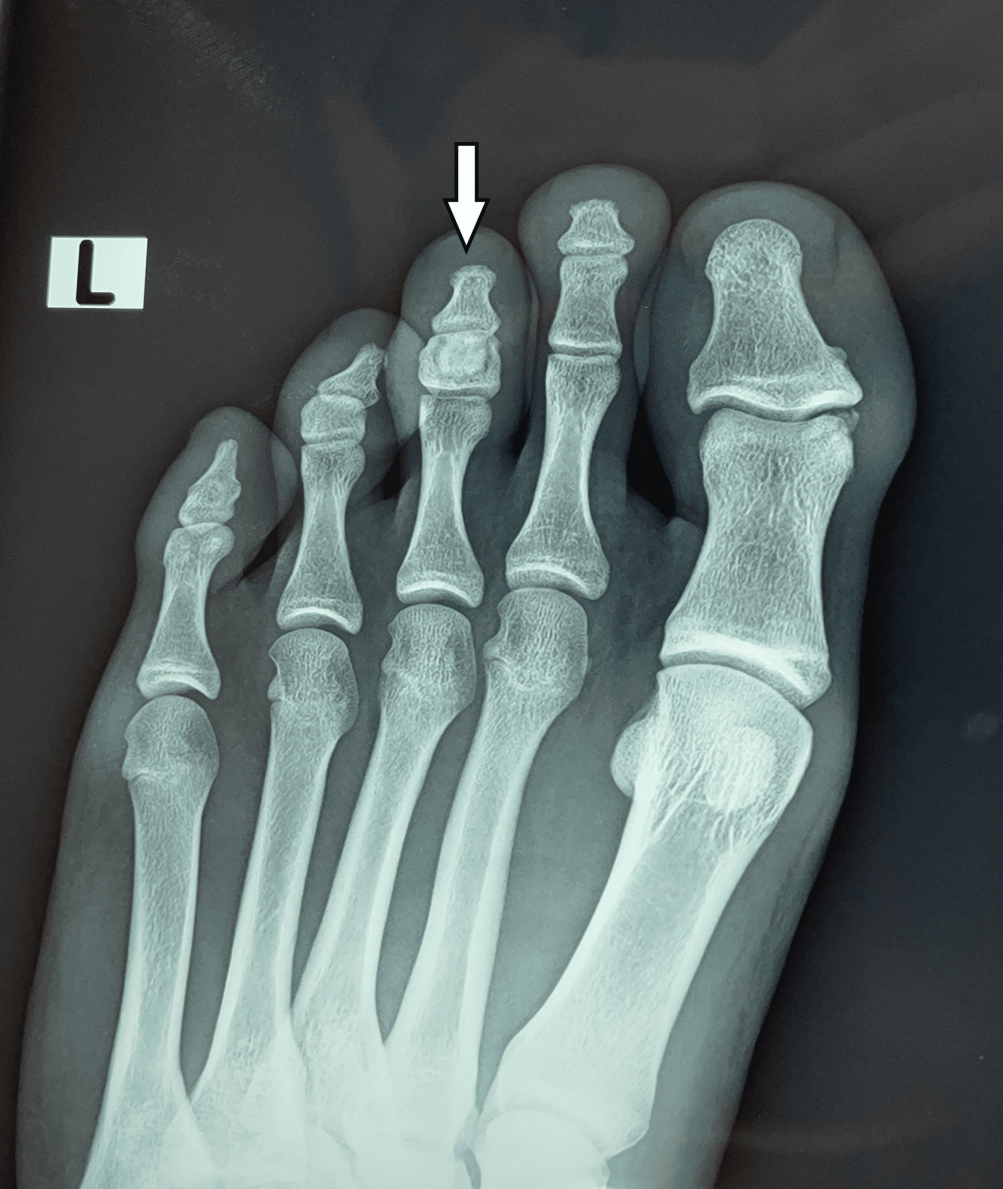
AP X-ray showing a central sclerotic lesion in the middle phalanx of the third toe, surrounded by a thin radiolucent zone

**Figure 3:**
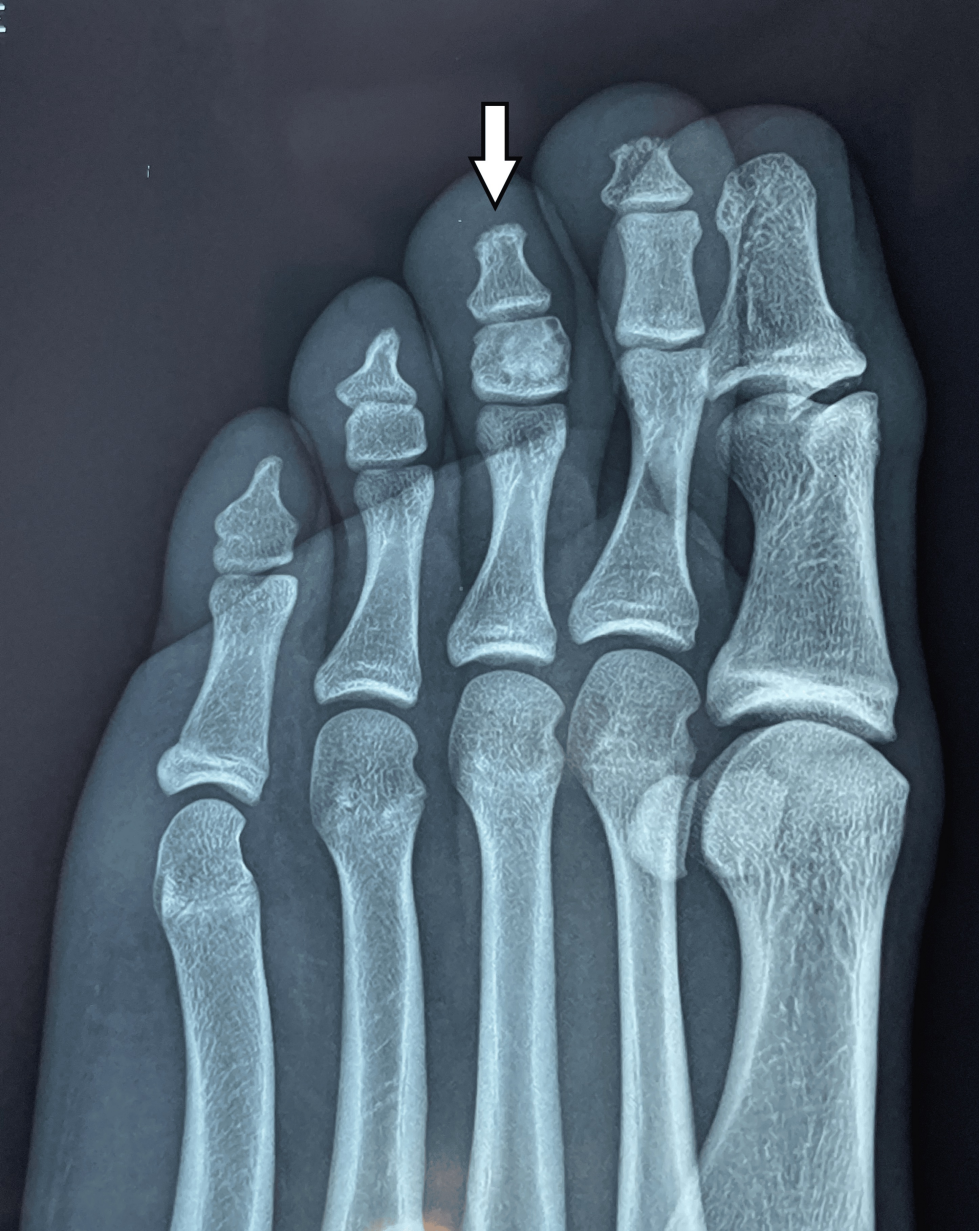
Lateral X-ray showing a central sclerotic lesion in the middle phalanx of the third toe, surrounded by a thin radiolucent zone

Examination of the feet revealed a distinct lesion affecting the medullary area of the middle phalanx of the third toe on the left foot. Irregular increased density occupied the entire phalanx, with a clear radiolucent zone surrounding the central sclerotic lesion. The bone cortices were intact, and the lesion was described as a bony island in the phalanx (Figure 2, Figure 3).

**Figure 4:**
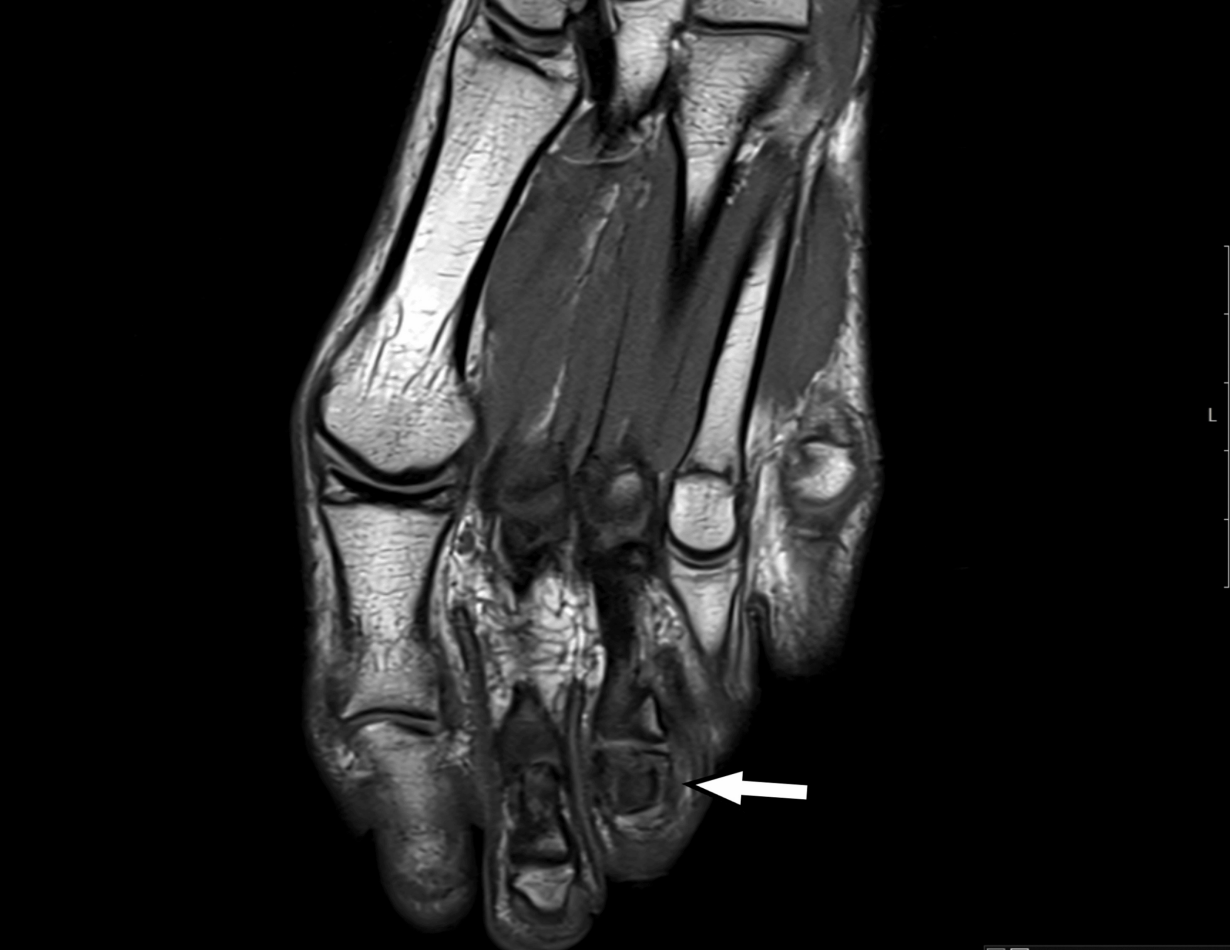
T1-weighted MRI showing an intermediate signal at the center of the lesion with low-signal intensity at the periphery

**Figure 5:**
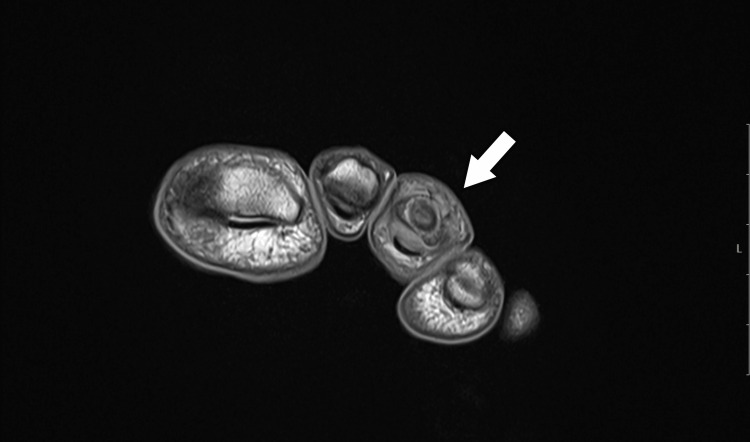
T1-weighted MRI showing an intermediate signal at the center of the lesion with low-signal intensity at the periphery

**Figure 6:**
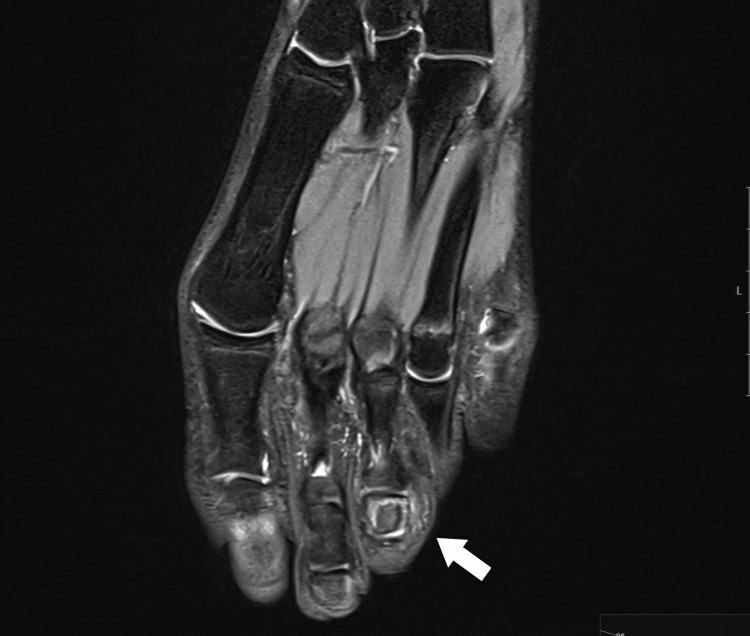
Axial T2-weighted MRI showing a centrally located high-intensity signal within the middle phalanx of the third toe, with a thin surrounding area of edema

**Figure 7:**
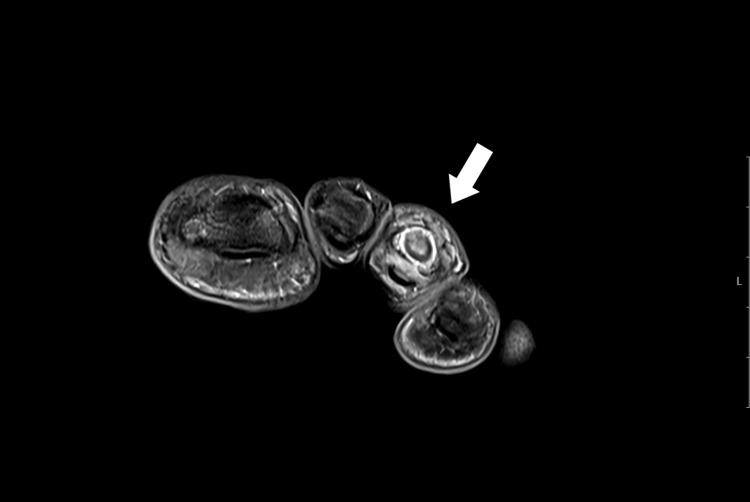
Coronal T2-weighted MRI showing a central high-intensity signal in the middle phalanx of the third toe, with a thin surrounding area of edema

An MRI examination was performed, revealing a lesion with intermediate to high signal intensity centered on the middle phalanx of the third toe, accompanied by peripheral bone marrow edema and thinning of the cortex on T2 coronal and axial imaging. T1 coronal and axial imaging showed an intermediate signal intensity lesion at the center of the phalanx, with low peripheral foci. Although the central nidus was not clearly visible, the lesion’s similarity to OO was evident due to the sclerosing bone within the cancellous bone (Figure 4, Figure 5, Figure 6, Figure 7).

**Figure 8:**
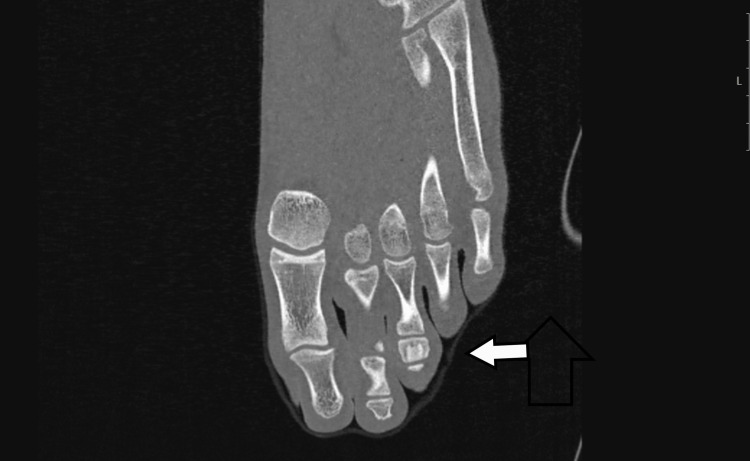
CT scan showing a centrally located sclerotic bone with a small radiolucent hypodense area in the transverse view

**Figure 9:**
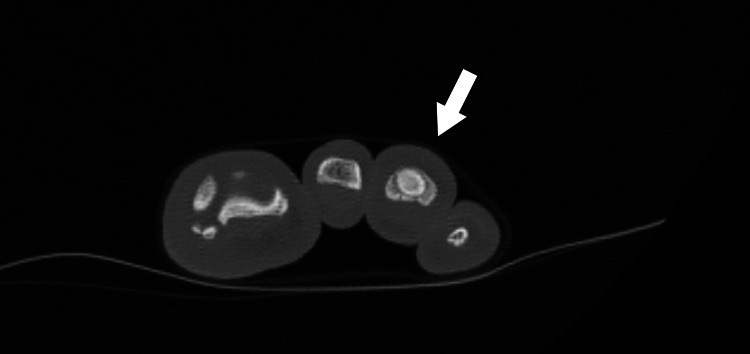
CT scan showing a centrally located sclerotic bone with a small radiolucent hypodense area in the transverse view

**Figure 10:**
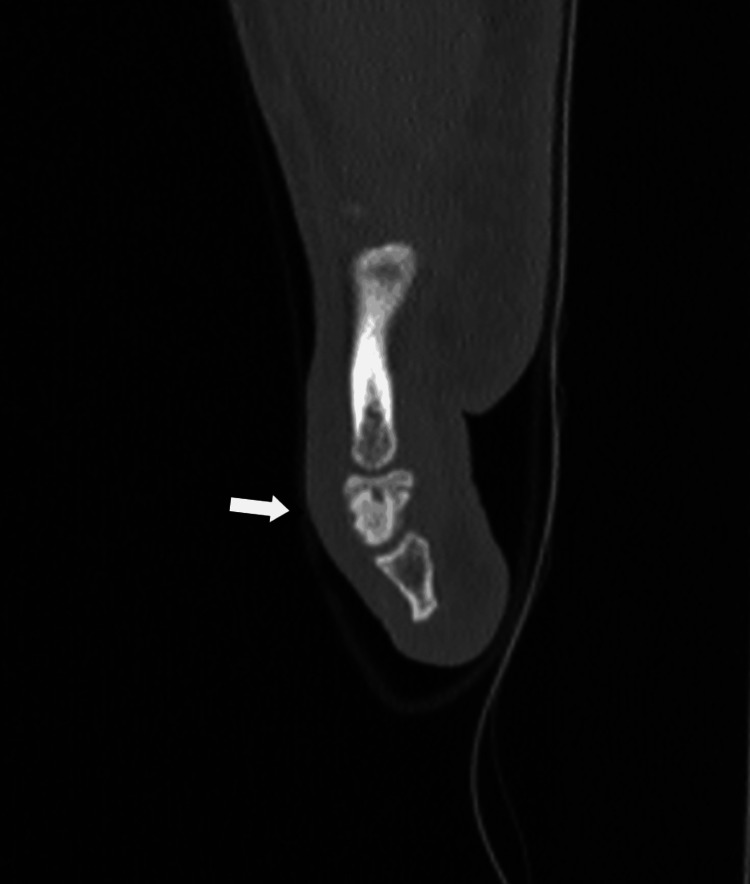
CT scan showing a centrally located sclerotic bone with a small radiolucent hypodense area in the transverse view

A CT scan, using 3D axial, sagittal, and coronal reconstruction, revealed sclerotic bone occupying the central area of the middle phalanx, surrounded by a hypodense area with a thinning, intact cortex. The central nidus was barely noticeable, appearing as a hypodense ovoid, primarily visible in the transverse view (Figure 8, Figure 9, Figure 10).

**Figure 11:**
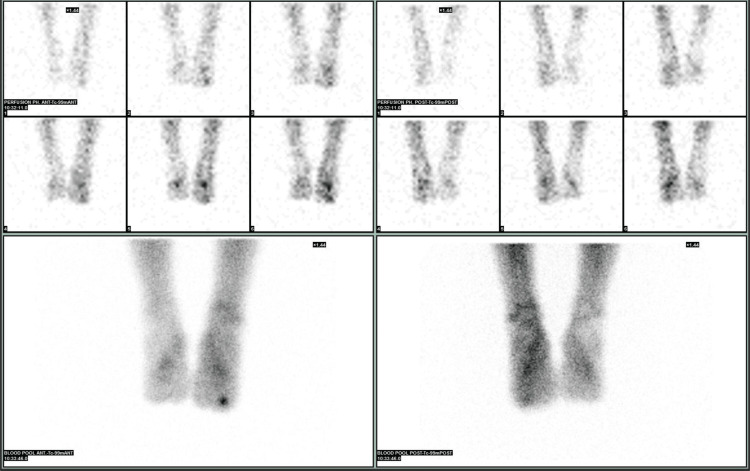
Increased uptake in all three phases of the bone scan for the middle phalanx of the third toe

**Figure 12:**
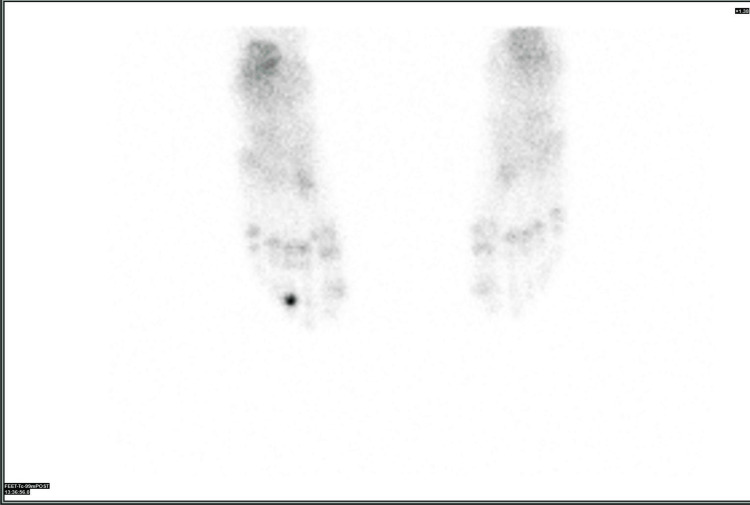
Increased uptake in all three phases of the bone scan in the middle phalanx of the third toe

The differential diagnosis included, in addition to OO, a sclerosing type of infection such as Garre’s osteomyelitis, a bony island, and an osteoblastoma. Bone scintigraphy was performed, showing typical Tc-99 uptake in all three phases of the central lesion in the toe, supporting the diagnosis of OO (Figure 11, Figure 12).

**Figure 13:**
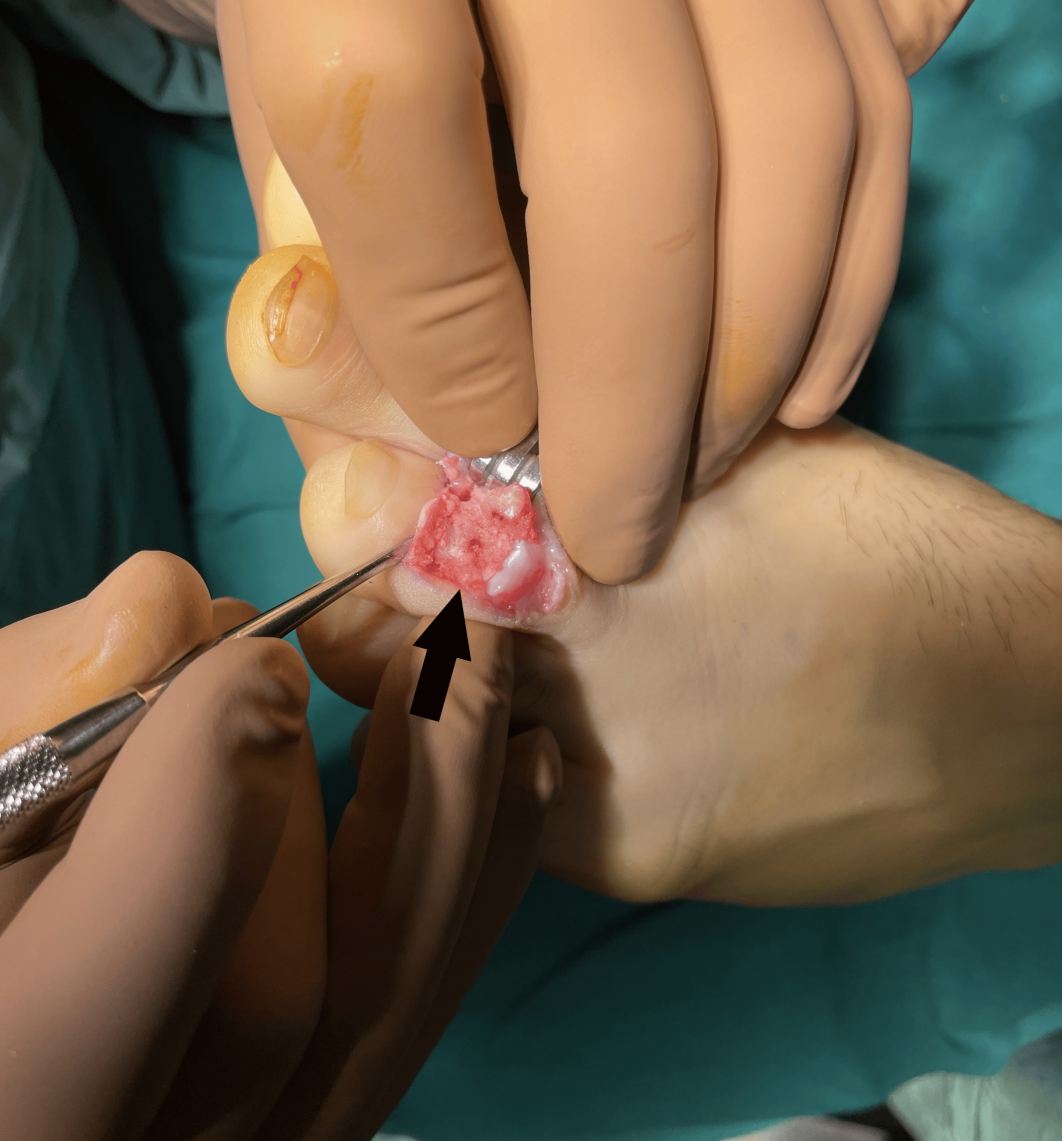
Intraoperative image showing the pink nidus at the center of the sclerotic bone

To achieve a definitive diagnosis, we performed an excisional biopsy to remove the entire sclerotic lesion. An L-shaped dorsal incision was made over the dorsum of the third toe, and after removing the skin flaps, we encountered diffuse granulomatous tissue over the bone cortices. A window was created in the dorsum of the middle phalanx by removing the dorsal cortical bone. Inside the phalanx, we found a hard sclerotic bone mass with no clear boundaries from the cortices, as the bone gradually transitioned from soft at the periphery to hard at the center. Upon opening the sclerotic bone mass, a central area of pink bone was observed, indicating the nidus of the OO (Figure 13).

**Figure 14:**
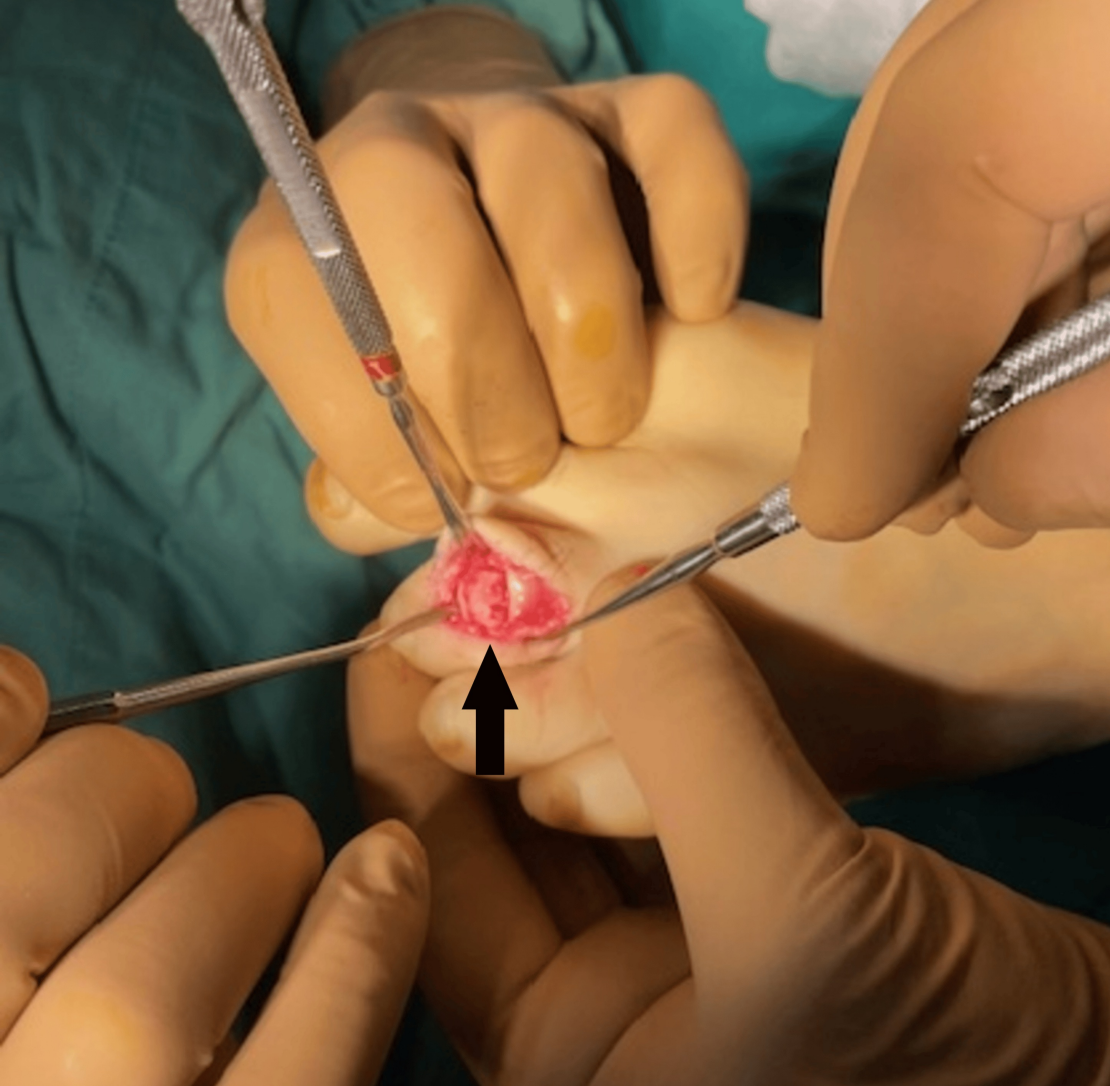
Restoration of the phalanx cortex using a cortical graft

The hardness of the central lesion was striking, raising concerns about the potential difficulties in inserting a bone trocar for biopsy and placing the RF electrode. After removing the sclerotic bone while preserving the articular surfaces, we filled the empty phalanx with cancellous and cortical grafts taken from the distal tibial metaphysis. The wound was then appropriately closed (Figure 14).

**Figure 15:**
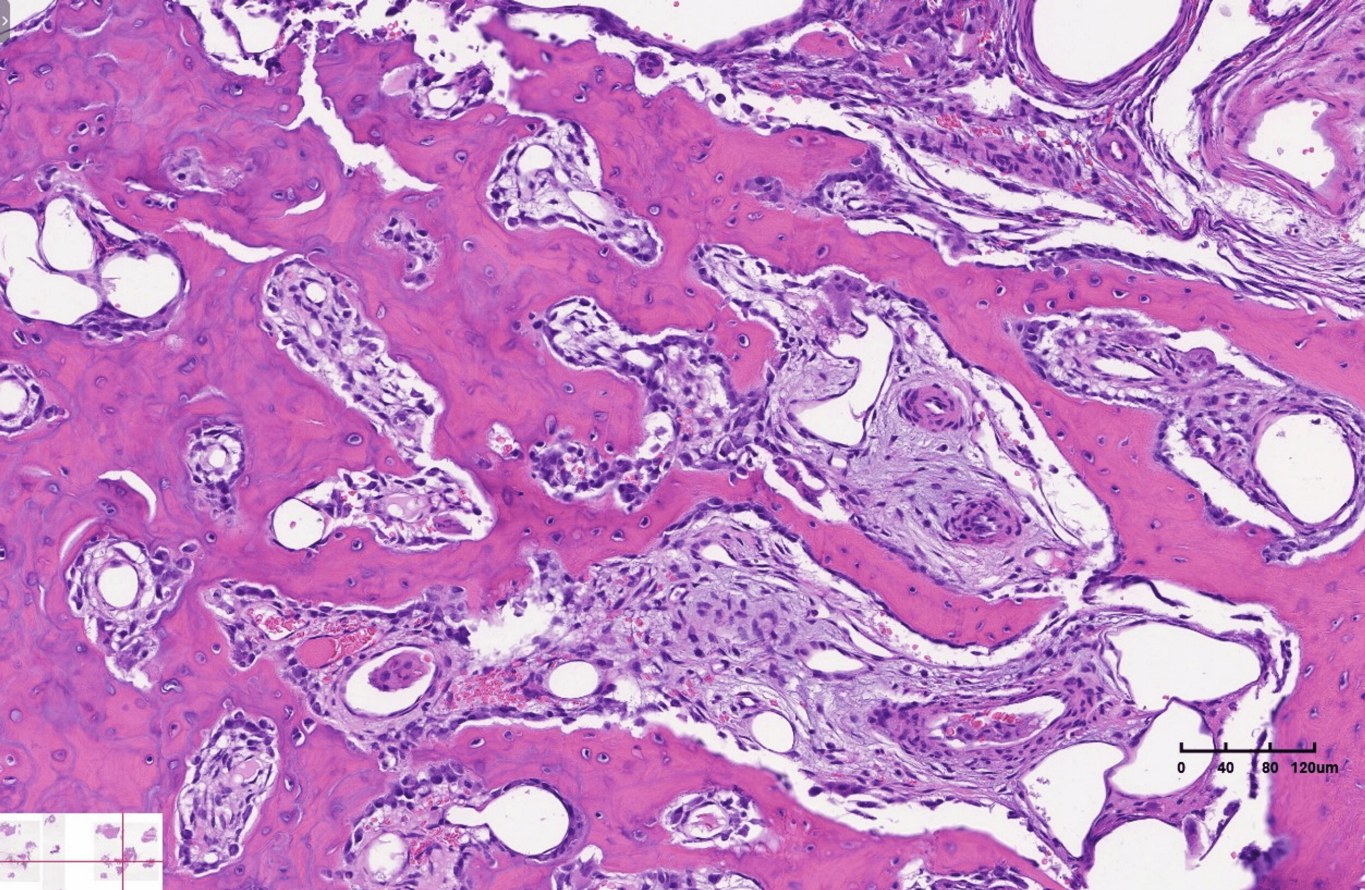
Numerous trabeculae of osteoid, thick and interlacing, with loose fibrous tissue and thin-walled vessels between them

The pathology confirmed the diagnosis of OO. Numerous, densely arranged, thick, and interlacing trabeculae of osteoid were observed. Prominent osteoblastic activity was present without osteocyte atypia. Between the osteoid trabeculae, small areas of loose fibrous tissue were seen, containing small, thin-walled vessels and a few osteoblastic-type multinucleated giant cells (Figure 15). The boy had an uneventful recovery.

## Discussion

OO affecting the toes is a rare occurrence, with few cases reported in the literature. Swelling is the predominant clinical symptom in these cases. While pain is the most common symptom of OO, typically exacerbating at night and relieved by NSAIDs, our patient reported only mild pain during football activities and did not experience night pain. Painless OO of the distal phalanx of the toe has been described by Haoudou et al. The reasons for the absence of pain in these cases remain unclear, with hypotheses suggesting a lack of nerve fibers, an absence of a rigid shell around the nidus, or the proximity of the lesion to the skin. However, these explanations lack substantial support [7-9].

In contrast, swelling of the toe can be mistaken for arthritis, leading to delayed diagnosis, as illustrated by Civino et al. [10]. Thiemann et al. published a case report of OO in the distal phalanx of the big toe, which clinically presented with macrodactyly [3].

Plain X-rays of our patient revealed increased medullary sclerosis. The lesion was typically located in the center of the phalanx, whereas OOs are usually found in the cortical areas of long bones. CT scans are considered the most reliable imaging modality for identifying the central nidus. In our patient, the primary feature was the sclerotic bone, and the central nidus was barely visible. Brodie’s sclerosing infection was included in the differential diagnosis. The most common finding in MRI is the presence of edema. MRI typically shows low intensity on T1-weighted images and increased intensity on T2-weighted images, with high contrast enhancement following gadolinium injection. It has been reported that in up to 35% of cases, the nidus may be obscured by surrounding edema. Small lesions can be difficult to detect on MRI, as the nidus signal often resembles that of the surrounding cortex [11,12].

MRI findings frequently suggest infection or rare types of soft tissue tumors. Castillo-Fortuño et al. proposed a diagnosis of a glomus tumor based on MRI findings of a pseudonodular subungual lesion affecting the distal phalanx of the second toe. Initial surgical exploration by dermatologists did not lead to a definitive diagnosis, but an excisional biopsy of the distal phalanx eventually confirmed OO [5].

Bellemans et al. reported a case of OO in the lesser toe of an 11-year-old boy, initially managed for arthritis. A needle biopsy was performed, but the insufficient tissue sample did not permit histological diagnosis. An excisional biopsy was later conducted, confirming the diagnosis [6].

Seo et al. reported an OO in the proximal phalanx of the great toe in a 13-year-old girl who presented with pain, swelling, and a burning sensation in the toe. Their investigation revealed clear OO findings on both MRI and CT scans, with subacute osteomyelitis and osteoblastoma included in the differential diagnosis. Interestingly, they also observed a greyish-red lesion during the excisional procedure, corresponding to the nidus of the OO [4].

Currently, OO is primarily treated with RF ablation, which is also our preferred method. CT-guided biopsy, when combined with RF ablation, has been reported to have less than a 50% success rate in confirming the diagnosis [12]. Jarolia et al. reported successful treatment of an OO in the first phalanx of the great toe with RF ablation, without pathological confirmation [13].

OO of the toes presents a diagnostic dilemma in nearly all reported cases. A definitive histopathological diagnosis is crucial for accurate diagnosis. In our case, the increasingly hard bone made it extremely difficult to place the needle for ablation accurately, as the central nidus was not visible on CT. The risk of fracturing the phalanx was significant. We successfully restored the phalanx size by filling the defect with cancellous bone, reconstructing the dorsal cortex with a piece of cortical bone, and preserving the articular surfaces.

## Conclusions

OO affecting the toes is an exceptionally rare occurrence. Our patient presented with a painless swelling of the toe. Radiological examinations, including X-rays, MRIs, and CT scans, revealed central calcification resembling a bony island, with the nidus being barely discernible. The bone scan showed positive uptake. We performed an open excisional biopsy, and pathology confirmed the diagnosis of OO in the middle phalanx of the third toe.
